# Systems metabolic engineering as an enabling technology in accomplishing sustainable development goals

**DOI:** 10.1111/1751-7915.12766

**Published:** 2017-07-11

**Authors:** Dongsoo Yang, Jae Sung Cho, Kyeong Rok Choi, Hyun Uk Kim, Sang Yup Lee

**Affiliations:** ^1^ Metabolic and Biomolecular Engineering National Research Laboratory Department of Chemical and Biomolecular Engineering (BK21 Plus Program) Institute for the BioCentury Korea Advanced Institute of Science and Technology (KAIST) Daejeon 34141 Republic of Korea; ^2^ BioInformatics Research Center KAIST Daejeon 34141 Republic of Korea; ^3^ BioProcess Engineering Research Center KAIST Daejeon 34141 Republic of Korea

## Abstract

With pressing issues arising in recent years, the United Nations proposed 17 Sustainable Development Goals (SDGs) as an agenda urging international cooperations for sustainable development. In this perspective, we examine the roles of systems metabolic engineering (SysME) and its contribution to improving the quality of life and protecting our environment, presenting how this field of study offers resolutions to the SDGs with relevant examples. We conclude with offering our opinion on the current state of SysME and the direction it should move forward in the generations to come, explicitly focusing on addressing the SDGs.

Systems metabolic engineering (SysME) is an enabling technology for optimizing cellular performance to produce better bioproducts to higher titres with higher productivities and yields. In particular, it has become essential in developing industrial microbial strains for the sustainable production of chemicals and materials. Combining synthetic biology, systems biology, evolutionary engineering together with traditional metabolic engineering, SysME upgrades the performance of living organisms far beyond their native capacity to produce industrially relevant compounds (Lee and Kim, 2015). In establishing the term biorefinery, metabolic engineering has set sail to contribute to the production of useful compounds – from bulk and specialty chemicals to polymers and materials – using renewable non‐food resources in hopes of ultimately replacing the conventional petroleum‐based industry, and addressing climate change and fossil resource depletion (Fig. 1). Rapid development of tools and strategies including omics technologies, computational modeling/simulation, and genetic engineering tools such as CRISPR/Cas and small regulatory RNAs has made SysME more powerful in developing microbial strains that will be used as cell factories for biorefineries. With pressing global issues arising in the 21st century, the United Nations (UN) announced 17 Sustainable Development Goals (SDGs). Such international effort is well aligned with recent movements from the World Economic Forum (WEF) that has selected SysME as one of the top 10 emerging technologies (https://www.scientificamerican.com/article/systems-metabolic-engineering-turns-microbes-into-factories/). The 17 SDGs formally accepted in September 2015 by the UN General Assembly is a set of measurable goals ranging from ending world poverty and hunger to combating climate change by 2030 (see Jang *et al*. in the same issue for more detail). In this short perspective, we discuss the roles of SysME in accomplishing the SDGs (words in bold below) that can be largely categorized into those related to ‘quality of human life’ and ‘environment’.

**Figure 1:**
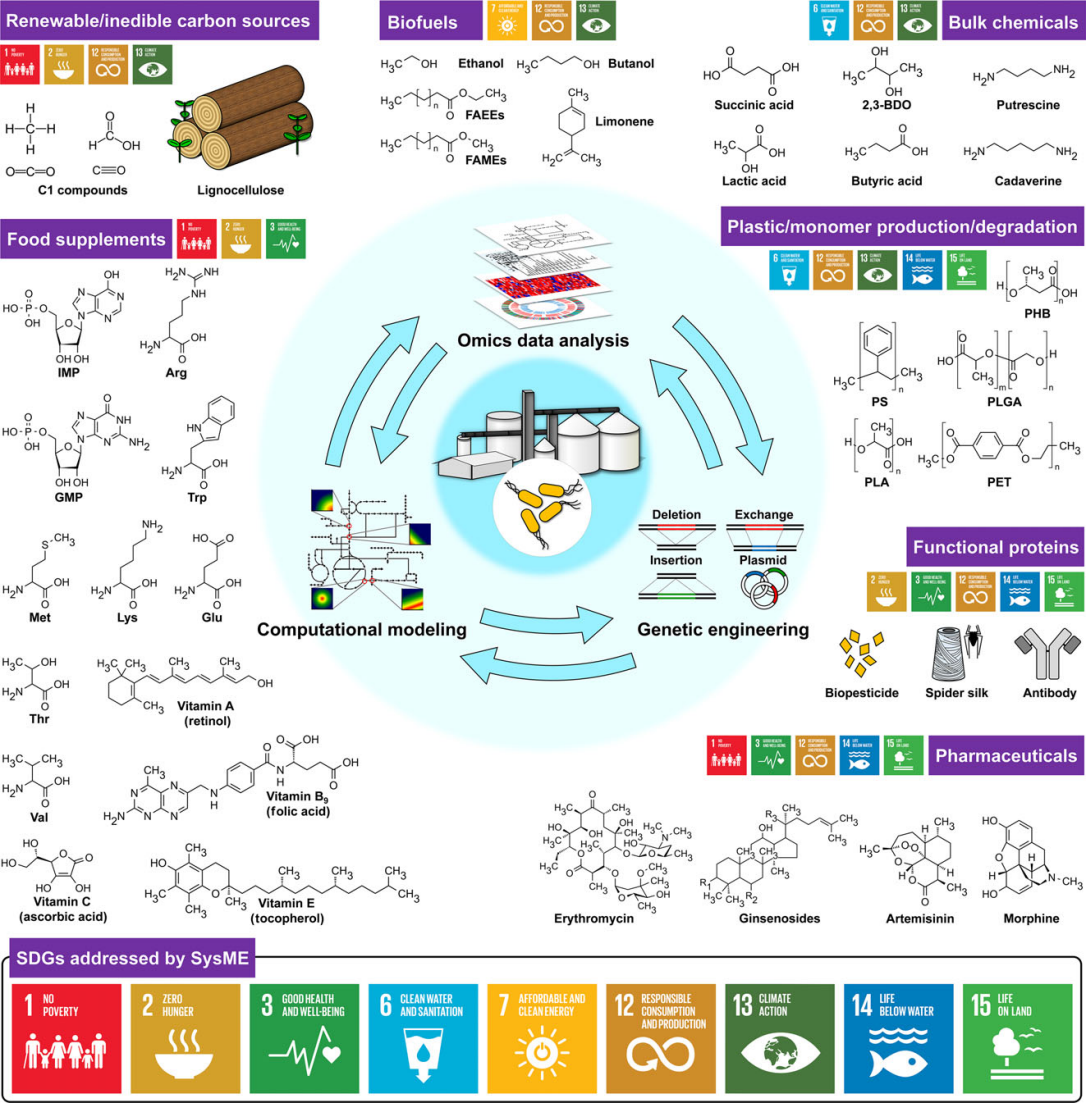
Example achievements of systems metabolic engineering (SysME) and their contributions to Sustainable Development Goals (SDGs). Abbreviations are as follows: 2,3‐BDO, 2,3‐butanediol; IMP, inosine monophosphate; GMP, guanosine monophosphate; Arg, arginine; Trp, tryptophan; Val, valine; Lys, lysine; Met, methionine; Glu, glutamic acid; Thr, threonine; PHB, poly(3‐hydroxybutyric acid); PS, polystyrene; PLGA, poly(lactate‐*co*‐glycolate); PLA, polylactic acid; PET, polyethylene terephthalate; FAMEs, fatty acid methyl esters; FAEEs, fatty acid ethyl esters.

In addressing **no poverty** (SDG 1) and **zero hunger** (SDG 2), SysME has been contributing to producing food and feed supplements (e.g., amino acids and nutraceuticals) through industrial fermentation of engineered microbes. History of fermentative amino acid production clearly showcases the contribution. An amino acid producer *Corynebacterium glutamicum* had been discovered, and its fermentation process established in hopes to end poverty and hunger in postwar Japan 60 years ago (Kinoshita, 2005). Today, *C. glutamicum* is a dominant microorganism engineered in industry for the production of many amino acids and their derivatives for their use in food and feedstock supplements (Becker and Wittmann, 2015). As traditional carbon substrates for such microbial fermentation processes have been in conflict with the supply of human food source (e.g., starch and sugar), methods to use non‐food biomass as carbon substrates are being developed through SysME (Bokinsky *et al*., 2011).

Other than food problems, **good health and well‐being** (SDG 3) can be achieved by providing strategies to treat diseases through SysME. A large portion of important drugs originate from plants, which suffer from their vulnerability to climate change and hence unstable supply. Of the most prominent examples is the production of artemisinin, an antimalarial drug, using an engineered yeast (Paddon *et al*., 2013). Other notable examples include the production of taxol (Ajikumar *et al*., 2010) and opioids (Galanie *et al*., 2015). Actinomycetes are an important resource for the production of secondary metabolites with various bioactivities including antibiotics, antifungal, anticancer and immunomodulation. SysME has been increasingly employed to allow more efficient design, control and optimization of these secondary metabolites (Weber *et al*., 2015), as showcased for erythromycin (Pfeifer *et al*., 2001; Zhang *et al*., 2010). These achievements have been made possible through large gene cluster cloning (Yuan *et al*., 2016), coordinated gene expression optimization (Na *et al*., 2013) and genome editing techniques (Wang *et al*., 2012) among many other SysME tools developed. Also, SysME strategies can be employed to perform system‐wide analyses of human metabolism and gene regulatory networks for drug target identification (Hur *et al*., 2017) and novel antimicrobial development through understanding host–pathogen interactions (Kim *et al*., 2010, 2011). Traditional oriental medicine can be modernized by systems biological analyses as multicomponent multitarget approaches will become increasingly important (Kim *et al*., 2015). Successful establishment of precision medicine and microbiome‐based health maintenance will also heavily rely on systems biology and SysME.

In addressing **responsible consumption and production** (SDG 12), SysME offers sustainable production of various chemicals and materials from renewable non‐food biomass (Lee *et al*., 2012; Lee and Kim, 2015). For instance, the scope of bacterial cellulose (BC) applications has been expanded from simple food industry to cosmetics, and biomedical and electronics industries, thanks to remarkable physical properties of BC (see Jang *et al*. in the same issue on BC). In addition, microbial natural polyesters polyhydroxyalkanoates (PHAs), and even non‐natural polymers including polylactic acid (PLA) and poly(lactate‐*co*‐glycolate) (PLGA) can be produced by one‐step fermentation of engineered bacteria developed with SysME (Jung *et al*., 2010; Park *et al*., 2012; Yang *et al*., 2013; Choi *et al*., 2016). These biodegradable polyesters can be used just like other polyesters, can be recycled, and once disposed, are biodegraded, which presents perfect carbon cycle for the environment. Other than producing biodegradable plastics, degradation of previously conceived ‘non‐degradable’ plastics might become possible, when needed, through SysME. With interesting discoveries of novel enzymes or strains that degrade polyethylene terephthalate (PET) (Yoshida *et al*., 2016) and polystyrene (Yang *et al*., 2015), the possibility of employing engineered microorganisms developed through SysME for degrading and/or recycling of plastic wastes might be realized.

Demand for freshwater is expected to increase due to ever‐increasing population and decreasing freshwater availability caused by climate change. As water scarcity is expected to jeopardize life and environment, our immediate action is needed to accomplish **clean water and sanitation** (SDG 6). Bio‐based production of chemicals and materials relies on the use of freshwater for the cultivation of microorganisms. To reduce the dependence of fermentation on freshwater, microorganisms that can grow well using seawater can be employed. As these halophilic microorganisms generally do not produce chemicals and materials of our interest with high enough efficiency, SysME will play an increasingly important role in upgrading their performance (Fu *et al*., 2014; Tan *et al*., 2014). Wastewater treatment can be enhanced by employing consortium of engineered microorganisms developed with SysME for more efficient regeneration of freshwater. When using such engineered microbial consortium in open field, SysME will also play important role in securing biocontainment to avoid the release of engineered microorganisms into nature (see below).

Currently, 80% of all energy comes from fossil fuels with only 14% from hydropower, biomass and other renewable resources (U.S. Energy Information Administration, 2017). SysME has contributed to the pursuit of **affordable and clean energy** (SDG 7) and **climate action** (SDG 13) in several different ways. SysME has been successfully employed for the development of microorganisms for the production of gasoline (Choi and Lee, 2013), biodiesel (Steen *et al*., 2010) and jet fuel (Renninger and McPhee, 2008) from renewable resources. The portfolio of chemicals and materials produced by microorganisms engineered through SysME is expanding at an unparalleled pace (Lee *et al*., 2012). One‐carbon (C1) compounds such as methane or CO_2_ abundant on earth have begun to be considered as potential alternative substrates through their conversion to formic acid (Bar‐Even, 2016), the simplest organic compound that can provide microbes with both carbon and reducing power for the production of various value‐added chemicals (Bar‐Even, 2016; Yishai *et al*., 2016). A bottleneck to using formic acid has been an assimilation step, but this has been addressed recently by developing a computationally designed enzyme that can now readily utilize formic acid (Siegel *et al*., 2015; Tai and Zhang, 2015). In a broader perspective, carbon‐fixing organisms such as algae and cyanobacteria have also been exploited through metabolic engineering to produce hydrogen (Beer *et al*., 2009), biofuels (Ducat *et al*., 2011; Lan and Liao, 2011) and polymers (Ducat *et al*., 2011).

SysME will also contribute to the preservation of **life on water** (SDG 14) and **life on land** (SDG 15), when engineered microorganisms are actively used in bio‐based economy. SysME can be employed to develop biocontainment system to avoid uncontrolled proliferation of the engineered organisms that can possibly perturb the natural ecosystem. Early biocontainment systems developed used auxotrophs in which only cells that have been supplied with exogenous metabolites (Steidler *et al*., 2003; Wright *et al*., 2013) or non‐natural amino acids (Mandell *et al*., 2015; Rovner *et al*., 2015) could grow. Of notable engineering feats in biocontainment systems are microbial kill switches with synthetic regulatory circuits developed recently (Chan *et al*., 2016).

Accomplishing the UN's SDGs will obviously require an integrated effort of science, technology, policy, public support, funding and regulation. SysME will not be the most important technology to address the SDGs, but will definitely contribute to achieving many of UN's SDGs as showcased above. With further advances in SysME, the list of successful examples will increase ranging from new drugs and better nutrition to sustainable chemicals and environmental protection. In the era of the fourth industrial revolution, SysME is expected to further evolve through the use of big data and artificial intelligence for designing high‐performance microorganisms and for developing safer and more robust therapeutics.

## Authors’ Contributions

SY Lee conceived the project. D Yang, JS Cho, KR Choi, HU Kim and SY Lee wrote the manuscript. All authors read and approved the final manuscript.

## Conflict of Interest

The authors declare that they have no conflict of interest.
